# eHealth and Web-Based Interventions for Informal Carers of People With Dementia in the Community: Umbrella Review

**DOI:** 10.2196/36727

**Published:** 2022-07-22

**Authors:** Bethan Naunton Morgan, Gill Windle, Rebecca Sharp, Carolien Lamers

**Affiliations:** 1 School of Human and Behavioural Sciences Bangor University Bangor United Kingdom; 2 School of Medical and Health Sciences Bangor University Bangor United Kingdom; 3 The University of Auckland Auckland New Zealand; 4 North Wales Clinical Psychology Programme Bangor University Bangor United Kingdom

**Keywords:** dementia, Alzheimer disease, informal, family, carers, caregivers, internet, online, technology, interventions

## Abstract

**Background:**

The prevalence of dementia is increasing, and there are many associated problems that family members face as informal carers, including emotional, physical, and financial difficulties. There are benefits for a person with dementia to live at home for as long as possible, and therefore, supporting their informal carers is crucial. The growing interest in supporting carers through internet-based interventions is evidenced by the volume of systematic reviews on this topic. It is now appropriate to systematically examine this body of work and provide an overview of the literature.

**Objective:**

This umbrella review aimed to identify the most effective internet-based intervention content and delivery method to support those caring for someone with dementia living in the community.

**Methods:**

PsycINFO, Web of Science, CINAHL, MEDLINE, Cochrane Library, and PubMed were searched for systematic reviews examining the effectiveness of web-based interventions for informal carers of people with dementia. A total of 3 reviewers extracted data and evaluated the quality of the papers. To ascertain the extent to which the systematic reviews reported on the same evidence, the proportion of overlap between their included studies was calculated. Qualitative research findings were extracted and reported.

**Results:**

A total of 21 papers were included in the study. The quality of the review papers was mainly rated as low to moderate, and 10% (2/21) of papers were of high quality. The findings suggest that multicomponent interventions were the most effective in supporting carers. These included combinations of cognitive behavioral therapy and relaxation strategies, educational resources, and online support groups. Interventions that were delivered on the web but included sessions with a personal element, such as telephone contact, showed the best results. When comparing the studies reviewed in all the review papers, a moderate overlap was noted. However, when comparing individual reviews with each other, they showed a high overlap of the included studies.

**Conclusions:**

Mixed delivery methods and intervention content showed the most effective results in supporting those caring for people with dementia. However, many papers do not separate the results for differing intervention contents or delivery; this needs to be considered when drawing conclusions. There was an overlap among the studies included in the reviews. This suggests a lack of current research on the effectiveness of web-based interventions for people caring for a person with dementia. There was also a lack of consistency in the outcome measures across all papers. Future studies can involve updating research on the effectiveness of these interventions while distinguishing between different intervention types, thus creating guidelines for the use of standardized measures to enable comparisons of intervention effects and improve the scientific quality of the overall research.

**Trial Registration:**

PROSPERO CRD42021241559; https://www.crd.york.ac.uk/prospero/display_record.php?RecordID=241559

## Introduction

### Background

Caring for someone with dementia is challenging emotionally and physically, and carers often need support in this role [1]. This is amplified when the person with dementia is a family member, and the carers are unlikely to have received any formal training in dementia knowledge or how to care for a person with dementia [2]. Supporting informal carers helps not only the carer but also the person with dementia by improving their quality of life and care, thus enabling them to remain at home for longer [1]. Informal carers are people who offer care and support to a person with dementia on a familial or friendship basis. An umbrella review of psychosocial interventions for informal carers of persons with dementia in 2017 identified 13 studies [1]. These included randomized controlled trials (RCTs) of interventions aimed at reducing stress, depression, and other mental health issues as well as physical health problems. They used a combination of educational resources on dementia, practical caring advice, and tips as well as peer support and psychotherapeutic methods to help carers adjust their way of thinking and cope with behavior changes related to dementia. Dickinson et al [1] concluded that multicomponent interventions consisting of educational, social, and therapeutic elements were most effective in improving the well-being of informal carers of persons with dementia. This suggests that carers need advice on caring for persons with dementia as well as for coping and managing their own emotions.

Differentiating between interventions targeting formal (professional) carers and those targeting informal carers is important. Formal carers will often be more experienced in caring for a person with dementia; hence, they will have some prior knowledge and training [2]. The relationship of a person with dementia with a formal carer is different from that with an informal carer, who often knows the person before the diagnosis, suggesting a more emotionally involved relationship. This was demonstrated when carers were asked to describe their reactions to aggressive behaviors from persons with dementia [3]. Informal carers said they devoted more time to the person with dementia and reduced outside contact, whereas formal carers gave practical ways to avoid behavioral episodes that did not isolate them from support systems [3]. This noted difference in approach could benefit from training and understanding of behavior, helping informal carers to consider alternative approaches.

Health care interventions have been moving toward becoming more technology based to combat the rising and unmaintainable health and social care costs because they require fewer staff and are able to reach more people at a similar cost [4]. This move has been accelerated by the COVID-19 pandemic, which forced several services to move to a web-based format, and many people learned how to use technology [5,6]. Technology-based interventions such as those administered on the web or by telephone are time- and cost-effective [1]. They are especially beneficial for carers in rural areas who would ordinarily have to travel to access such services [7]. Rurality may also cause difficulties in accessing the internet [5,6]; however, the number of people in the United Kingdom with access to the internet is consistently increasing [8]. Web-based interventions specifically use the internet, such as an educational website or a peer support forum. Technology-based interventions developed before 2000 were mainly administered by CD-ROM or DVD [4], and for the purpose of this review, they are not included in the definition of web-based intervention.

Web-based interventions that are based solely on education and do not have a *live* element, such as a video call, can be more convenient for busy carers to access at any time. They also require less bandwidth to run [9]; therefore, interventions without video calls will be accessible to more carers. However, not having the engagement and accountability of speaking to another person may reduce adherence to web-based interventions [10] and reduce personal contact.

Technology-based interventions may not be appropriate for all carers because of a range of factors, including age [2]. Spouse or sibling carers of persons with dementia are often older than those caring for a parent with dementia, resulting in a large age range for carers of persons with dementia. However, the number of adults aged ≥75 years in the United Kingdom who use the internet has increased by 26% between 2011 and 2019 [8], with a probable further increase since the COVID-19 pandemic. Given these rapidly evolving developments, it seems timely to review the evidence for web-based support for carers of persons with dementia by conducting an umbrella review of published systematic reviews of web-based interventions.

An umbrella review is a relatively new tool used for evidence synthesis [11]. The method was developed in response to the growing number of systematic reviews being published [11,12]. It is a systematic review of reviews that provides an overview of the information available on a subject [12]. It is often broader in scope than a systematic review and offers a summary that may be useful for policy makers [11,12]. This method of data synthesis was selected after a brief literature search revealed several systematic reviews focusing on the effectiveness of web-based interventions for informal carers of persons with dementia.

### Aims of This Review

This umbrella review aimed to synthesize systematic reviews of web-based interventions for informal carers. It will (1) identify types of web-based interventions that have been developed for informal carers of persons with dementia in the community and (2) report on which types are most effective in supporting carers of persons with dementia.

This umbrella review summarizes the topic by using a narrative approach. This may detect gaps in the topic and allow identification of effective methods for future interventions aimed at supporting informal carers of persons with dementia living in the community and enabling persons with dementia to live at home for longer.

### Research Question

The research question for this review was as follows:

1. “What types of web-based interventions have been developed for informal carers of persons with dementia living in the community?”

This was further developed into a subquestion:

2. “What are the types of web-based interventions that are most effective in supporting informal carers of persons with dementia living in the community?”

## Methods

### Overview

The methods used in this umbrella review were based on the guidelines from the Joanna Briggs Institute (JBI) [12]. The research question, search strategy, and inclusion criteria were developed before conducting the search. This protocol was registered with PROSPERO (International Prospective Register of Systematic Reviews; registration number CRD42021241559).

### Inclusion Criteria

The inclusion criteria were generated using the population, concept, and context guide as suggested by JBI [12]:

1. P—The population were informal carers of persons with dementia (eg, family members, friends, and neighbors that identified themselves as carers. There were no restrictions on the amount of time spent caring). Paid carers were excluded from this review.
2. C—The concept involved web-based interventions, which included psychosocial, educational, and therapeutic interventions administered on the web. This review focused specifically on web-based interventions owing to the convenience of being able to access them at any time compared with telephone or face-to-face interventions, which are more time sensitive. Therefore, telephone-only interventions, prerecorded videos, and face-to-face interventions were excluded.
3. C—The context for the review was carers for persons with dementia who were living at home. Carers caring for a person residing in a care home or hospital were excluded from the review, as they would usually include formal carers. Carers in the community, either adjusting to their role or managing ongoing stressors from offering regular care, may require support in adjusting to the role of a carer [13,14]. They may also require web-based interventions that they can access without leaving the house, so that they do not need to make alternative care arrangements to physically access supportive interventions [15]. Carers of people in care homes were excluded as although they continued to care for the person with dementia, their needs were conceptualized as different compared with those managing the care for persons with dementia living in the community.

Only systematic reviews and meta-analyses of web-based interventions were included in this study. We refer to systematic reviews as review papers and papers that they reviewed as study papers. Only reviews in English were included, but date limits were not imposed, as remote interventions before 2000 were mainly administered by CD-ROM or teleconferencing [16] and would not be classified as web-based interventions, and therefore, they were excluded.

### Search Strategy

#### Overview

The following databases were searched: PsycINFO, Web of Science, CINAHL, MEDLINE, Cochrane Library, and PubMed, as these contain papers from health, social care, and psychological perspectives. The reference lists of review papers were also manually searched, and furthermore, experts were consulted regarding the reviews to be included.

**Figure 1:**
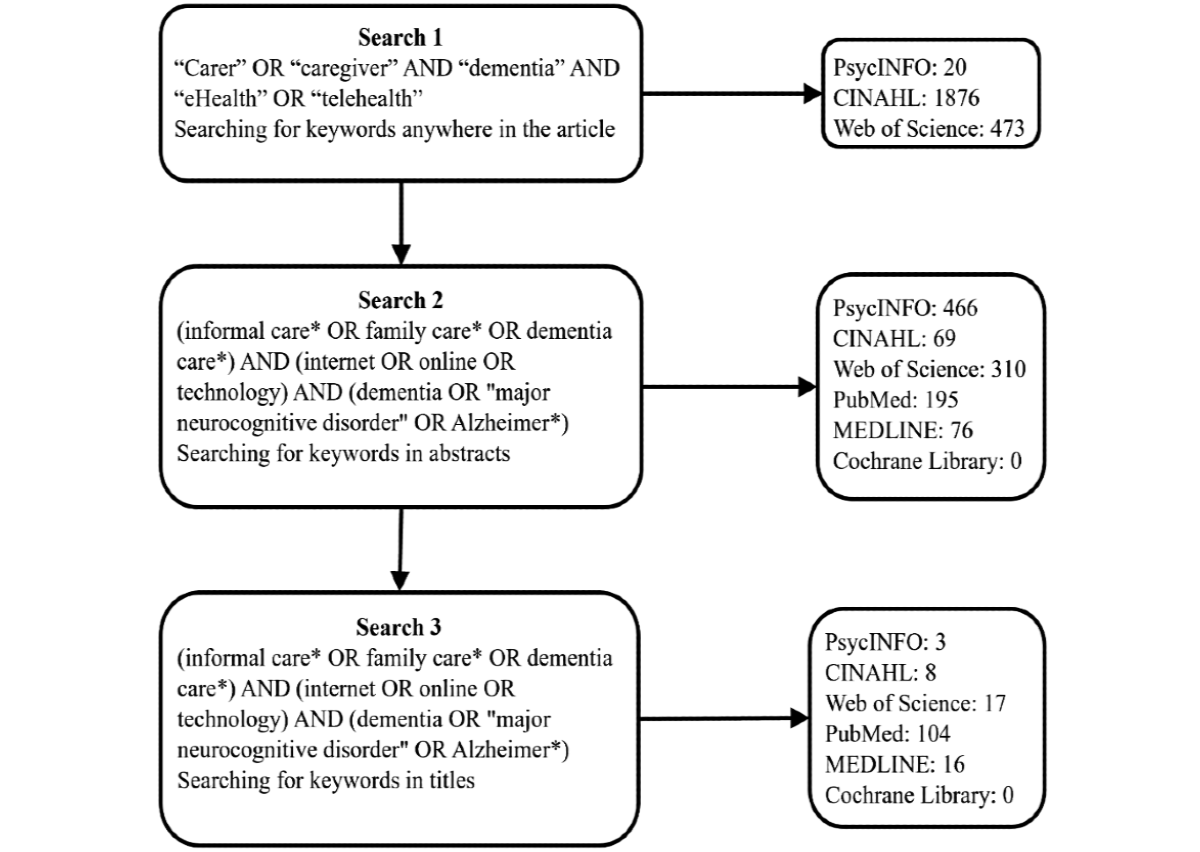
Keyword search terms and number of results for the 3 database searches.

In total, 3 pilot searches were performed to refine the search criteria, focusing on different keywords in different parts of the papers, which provided a better understanding of the literature to create a concise and comprehensive search strategy (Figure 1). This removed irrelevant papers, resulting in a satisfactory number of review papers that addressed the research question.

#### Search 1

The first search looked for the keywords anywhere in the papers, including the title, abstract, and text (Figure 1). The terms “dementia,” “major neurocognitive disorder,” and “Alzheimer*” were selected as they would identify papers focusing on a range of dementia subtypes including vascular dementia, dementia with Lewy bodies, and Alzheimer disease. The option to search for systematic reviews only was selected for each database. This search found 2369 review papers with many being irrelevant, containing interventions for carers of conditions other than dementia. This search was conducted only on the first 3 databases because of the large number of papers that were unrelated to the review. The words eHealth and telehealth were found to be more focused on physical health, such as blood pressure monitoring; consequently, they were replaced in the second search with more specific terms, that is, internet, on the web, and technology.

#### Search 2

The second search included more variations of the search terms used in the first search and focused on locating these only in the abstracts (Figure 1). This resulted in fewer papers (1116 in total); however, many papers focused on conditions other than dementia such as cancer.

#### Final Search

The third search used the same search terms, but these had to be included in the titles of the review papers, resulting in 148 results from 6 databases (Figure 1). Owing to the number and relevance of the results from the third search, this was the final search strategy used for the review. The strategy was adjusted for each database to use variations of the search that were functional. The final search was conducted on October 1, 2021. A further 3 papers were included based on the suggestion of the second reviewer.

### Study Selection

The results were downloaded from the databases and imported into the RefWorks (ProQuest LLC) web-based reference manager. The inbuilt tool was used to remove duplicates, and then, a manual search removed the remaining duplicates that were not identified by RefWorks, which resulted in the removal of 45 papers.

The lead author (BNM) reviewed the titles and abstracts of all 106 review papers to ensure that they met the inclusion criteria, leading to the exclusion of 75 papers. The full texts of 31 papers were assessed by the lead author (BNM), which led to the exclusion of 10 papers. To ensure that the eligibility criteria were applied consistently, coauthors (GW and CL) independently assessed 50% (5/10) of the excluded papers each. These papers were randomly allocated to the coauthors using a random number generator. The inclusion criteria were clear, and there was no disagreement regarding the exclusion of the papers.

Interventions aimed at formal (professional) carers in a care home or hospital setting were excluded, as this review focused on interventions for informal carers. However, several of the review papers included data collected from both formal and informal carers, which were still included as the exclusion of the papers would result in the loss of relevant data.

### Methodological Quality

The quality of the reviews was assessed using the A Measurement Tool to Assess Systematic Reviews (AMSTAR) 2 [17] quality assessment tool. This is an updated version of the AMSTAR tool [18], and it measures the methodological quality of systematic reviews. There are 16 items assessing the inclusion of systematic review methods that are considered high quality (Table 1). The AMSTAR 2 tool is not intended to rate the quality of a review paper as a whole, and it is advised to consider the impact of each individual item to provide a rating of overall confidence in the results [17]. Ratings range from critically low to high and are produced using the web-based assessment tool. The scores are dependent on the answers to 7 critical items presented in Table 1. A high-quality rating indicates <1 critical weakness in a review paper, moderate rating indicates >1 noncritical weakness, low rating indicates 1 critical weakness, and critically low rating indicates >1 critical weakness. All reviews were assessed for quality by the lead author (BNM), and the second and third authors (GW and RS) assessed 29% (6/21) of the papers each, resulting in 57% (12/21) of the reviews being assessed by 2 authors. The fourth author (CL) was consulted to reach a consensus in the case of any disagreement.

**Table 1:** A Measurement Tool to Assess Systematic Reviews (AMSTAR) 2 questions, responses, and “critical” items.

AMSTAR 2 questions	Responses	Critical domain
Did the research questions and inclusion criteria for the review include the components of PICO^a^?	Yes or no	No
Did the report of the review contain an explicit statement that the review methods were established before the conduct of the review, and did the report justify any significant deviations from the protocol?	Yes or partial yes or no	Yes
Did the review authors explain their selection of the study designs for inclusion in the review?	Yes or no	No
Did the review authors use a comprehensive literature search strategy?	Yes or partial yes or no	Yes
Did the review authors perform study selection in duplicate?	Yes or no	No
Did the review authors perform data extraction in duplicate?	Yes or no	No
Did the review authors provide a list of excluded studies and justify the exclusions?	Yes or partial yes or no	Yes
Did the review authors describe the included studies in adequate detail?	Yes or partial yes or no	No
Did the review authors use a satisfactory technique for assessing RoB^b^ in individual studies that were included in the review?	For RCTs^c^—yes or partial yes or no or includes only NRSI^d^; for NRSI—yes or partial yes or no or includes only RCTs	Yes
Did the review authors report on the sources of funding for the studies included in the review?	Yes or no	No
If meta-analysis was performed, did the review authors use appropriate methods for statistical combination of results?	For RCTs—yes or no or no meta-analysis conducted; for NRSI—yes or no or no meta-analysis conducted	Yes
If meta-analysis was performed, did the review authors assess the potential impact of RoB in individual studies on the results of the meta-analysis or other evidence synthesis?	Yes or no or no meta-analysis conducted	No
Did the review authors account for RoB in primary studies when interpreting or discussing the results of the review?	Yes or no	Yes
Did the review authors provide a satisfactory explanation for, and discussion of, any heterogeneity observed in the results of the review?	Yes or no	No
If they performed quantitative synthesis, did the review authors carry out an adequate investigation of publication bias (small study bias) and discuss its likely impact on the results of the review?	Yes or no or no meta-analysis conducted	Yes
Did the review authors report any potential sources of conflict of interest, including any funding they received for conducting the review?	Yes or no	No

^a^PICO: population, intervention, control group, outcome.

^b^RoB: risk of bias.

^c^RCT: randomized controlled trial.

^d^NRSI: nonrandomized studies of intervention.

### Corrected Covered Area

Review papers on similar topics may include the same studies in their reviews. Overlap of individual studies in systematic reviews can mask a lack of current research on a given topic. If new systematic reviews are being conducted, it can give a false impression of new evidence. To address the extent of overlap, the corrected covered area (CCA) [19] measure was used. The CCA was developed to compare the overlap of studies reviewed in meta-analysis reviews. Although this review was of systematic reviews, the CCA index was used to demonstrate the overlap of studies in the review papers. The CCA was calculated by multiplying the number of index publications by the number of review papers and then subtracting the number of index publications. This was then divided by the frequency of repeated studies. Index publications are the number of primary studies in the review papers, so that they are only counted once, disregarding any repeats in other review papers. A high CCA score indicates a high percentage of overlap.

The CCA measurement has been described as a “promising” measure of overlap; however, it is easily skewed by the inclusion of a single review containing many index publications [20]. To compensate for this potential skew, 5 steps were recommended: create a citation matrix (Multimedia Appendix 1 [15,16,21-39]), calculate the total CCA, calculate the CCA for reviews with high overlap, examine the topic areas for differences in date or samples, and discuss the potential implications of the overlap and report on similarities and differences in outcomes.

### Data Extraction

A table for data extraction was created based on the JBI [12] guidelines for conducting umbrella reviews. The data extracted included authors, date, country in which the study was undertaken, number of studies reviewed, population or demographics, sample sizes, intervention details (content and delivery), measures, aims, results, and key findings related to the research question.

Of 21 review papers, 3 (14%) [21-23] included data from both face-to-face interventions and technology-based interventions. The results from these 2 interventions were reported separately in each review, so only the data from the technology-based interventions were extracted (refer to column S in Multimedia Appendix 2 [15,16,21-39]). However, the definition of “technology-based” interventions varied among some of the review papers. For example, a study by Chang [40], which looked at a cognitive behavioral therapy–based intervention that was delivered by video and telephone, was classified as a technology-based intervention in the reviews by Deeken et al [24], Jackson et al [25], Lucero et al [26], and Waller et al [27] but not by Thompson et al [21]. The rationale for Thompson et al [21] classifying the study by Chang [40] as face-to-face was not clear. Consequently, the data from the study by Chang [40] were extracted from the reviews that defined it as a technology-based intervention [24-27].

Of 21 review papers, 3 (14%) included data from both formal and informal carers. Hopwood et al [28] included 40 studies, 3 (8%) of which were a mixture of formal and informal carers. Etxeberria et al [29] primarily incorporated informal carers, but 2 studies included formal carers. Pleasant et al [16] included 9 studies examining formal and informal carers. However, the results of these studies could not be separated from those of informal carers. As only 11.8% (14/119) of studies included both formal and informal carers, they were included in the data synthesis.

To evaluate the interrater reliability of the data extraction, the second and third reviewers (GW and RS) evaluated 57% (12/21) of the total review papers. Any disagreements were resolved by the fourth reviewer (CL).

### Data Synthesis

In line with the JBI [12] framework for conducting umbrella reviews, the data were presented as a summary of the synthesized results with no further analysis. The extracted data were used to populate a table of study characteristics (Multimedia Appendix 2) and a table summarizing the evidence. The framework states that the summary table should be presented visually, showing effective interventions, mixed results, and no significant improvements. Although the term “major neurocognitive disorder” was introduced in the Diagnostic and Statistical Manual of Mental Disorders, Fifth Edition [41], to help counter the stigma associated with the term “dementia,” research and health care settings continue to use the term dementia. The key terms used in the systematic search included both “dementia” and “major neurocognitive disorder”; however, we found that most of the papers used the term “dementia,” and consequently, for the purposes of this review, we use the term “dementia” throughout.

## Results

**Figure 2:**
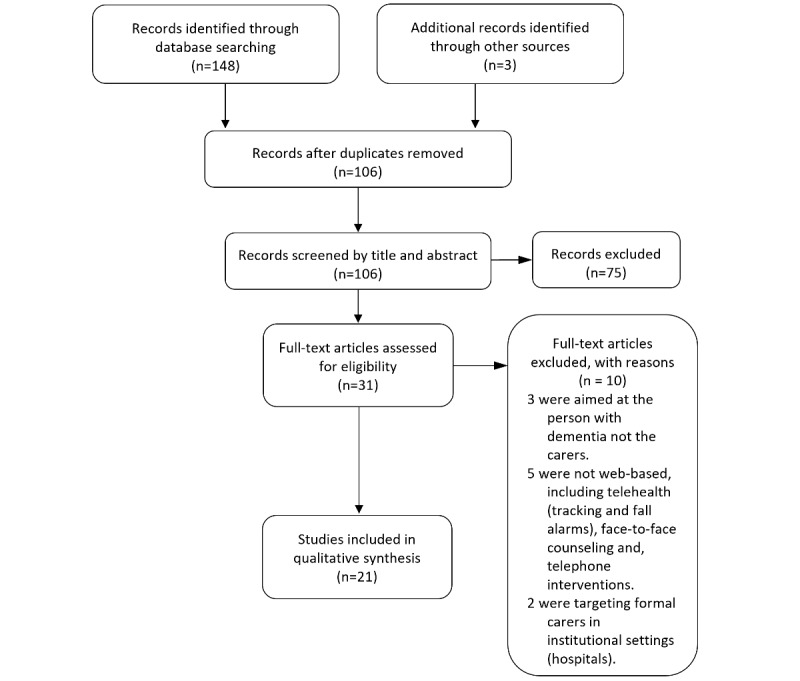
PRISMA (Preferred Reporting Items for Systematic Reviews and Meta-Analyses) diagram of the selection process.

The review process is illustrated in Figure 2. A total of 21 review papers met the inclusion criteria and were included for data synthesis.

### Methodological Quality

After reviewing the papers separately, there was agreement on 83% of the AMSTAR 2 [17] scores. Any disagreements were resolved by the third author. Of the 21 reviews included, 2 (9%) were rated as high quality, 9 (43%) were rated as moderate quality, 2 (9%) were rated as low quality, and 8 (38%) were rated as critically low quality (Multimedia Appendix 2). This suggests that despite the number of papers on a similar topic, only 2 contained the recommended depth. The most common issues were a lack of bias assessment and not having a protocol in place before conducting the search, which were suggested to be because of time constraints.

### CCA Scores

The total CCA score showed a 7% overlap, which is considered a moderate overlap; <5% is low overlap, and >15% is high overlap [19]. The CCA matrix (Multimedia Appendix 1) was used to determine reviews with a high overlap so that the CCA scores could be calculated for them. Individual CCA scores were calculated by comparing each of the 21 reviews with each other (Multimedia Appendix 1). The results showed that 26% of the CCA scores were high, 50% were moderate, and 24% were low. The highest overlap was 56%, which was between those reported by Zhao et al [30] and Egan et al [31].

### Characteristics of the Included Reviews

The total number of research studies included across the review papers was 119 (50 of these were included more than once). The number of studies included in each review paper ranged from 4 to 40. The research designs varied within each review paper and included mixed methods, RCTs, and pre- and postintervention measures.

### Participants

The sample sizes varied from a pre-post intervention study of 4 [28] to an RCT of 1222 participants [32]. The participants included mainly informal carers; 29% (6/21) review papers included a mix of formal and informal carers, and 14% (3/21) review papers also looked at the intervention effects on persons with dementia (Multimedia Appendix 2). There was an umbrella term of “dementia carers” being used to describe formal and informal carers for people with Alzheimer disease, vascular dementia, and early onset dementia; however, 57% (12/21) of the reviews did not describe the type of dementia that the care recipient had. Furthermore, 52% (11/21) reviews included more detailed information on the participants [15,24,26,27,29,31-35,42], including their nationalities. The countries in which the studies were conducted were reported in 52% (11/21) reviews. In addition, 52% (11/21) reviews reported the gender of carers. Participants were mainly female; however, 1 study [43] that was included in 3 of the reviews specifically looked at male carers [24-26]. Of the 21 reviews, 8 (38%) reviews described age of the carers, which ranged from 18 to 88 years.

### Interventions

Interventions were delivered using technology, including web, telephone, DVD, or a combination of these (Multimedia Appendix 2). The duration of the interventions varied from having 24-hour access to websites to 6-minute telephone sessions.

The content also varied; however, psychoeducation, psychotherapy, and social support were the most common types of interventions (Multimedia Appendix 2). Psychoeducational interventions provided information on caring and dementia to the carer. This included information from web-based encyclopedias, practical caring advice, classes with homework, web-based question-and-answer sessions with nurses, and web-based quizzes. The psychotherapy interventions contained elements of cognitive behavioral therapy to help carers manage their emotions and behavior, including cognitive restructuring, relaxation, and “telephone counseling.” The interventions offering social support used peer support via web-based groups, individual phone calls, and voice messages, facilitating carers to have contact with people in similar situations. Social support interventions have offered advice and coping strategies; however, this was from peers, and hence, it was more informal. At least one of these 3 intervention types was found to be present in all of the studies, often more than one, which is referred to as a multicomponent intervention.

### Outcomes of Review Papers

Outcomes differed between studies, with depression, anxiety, burden, and self-efficacy being most commonly measured. However, the measures used to assess these outcomes varied between the reviews. Waller et al [27] reported 5 different measures for depression (Table 2), and Jackson et al [25] reported 5 measures for burden. This variation in measures made pooling results difficult [31]; however, 7 reviews reported a meta-analysis of the results [15,21,23,24,29,30,36].

**Table 2:** Depression measures used in the 21 review papers.

Authors	Depression measures	Number of depression measures
Leng et al, 2020 [15]	CES-D^a^, BDI-II^b^, and PHQ-9^c^ questions	3
Deeken et al, 2019 [24]	CES-D, BDI, GDS^d^, and BSI^e^ depression subscale	4
Hopwood et al, 2018 [28]	CES-D and PHQ-9	2
Boots et al, 2014 [38]	CES-D	1
Egan et al, 2018 [31]	CES-D and BDI-II	2
Jackson et al, 2016 [25]	CES-D, BDI, and GDRS^f^	3
Lucero et al, 2019 [26]	NR^g^	NR
Pleasant et al, 2020 [16]	CES-D, BDI-II, and GDS	3
Scott et al, 2016 [36]	CES-D and BDI-SF^h^	2
Waller et al, 2017 [27]	CES-D, BDI-II, PHQ-9, GDS, and SDS^i^	5
Zhao et al, 2019 [30]	CES-D and BDI-II	2
Etxeberria et al, 2020 [29]	CES-D, BDI, PHQ, and SDS	4
Frias et al, 2020 [22]	CES-D	1
Godwin et al, 2013 [32]	CES-D	1
Kishita et al, 2018 [23]	CES-D and BDI	2
Klimova et al, 2019 [33]	CES-D	1
Parra-Vidales et al, 2017 [37]	CES-D and BDI	2
Powell et al, 2008 [39]	NR	NR
Lee, 2015 [34]	CES-D	1
Thompson et al, 2007 [21]	NR	NR
McKechnie et al, 2014 [35]	CES-D	1

^a^CES-D: Center for Epidemiologic Studies Depression Scale.

^b^BDI-II: Beck Depression Inventory-II.

^c^PHQ-9: Patient Health Questionnaire-9.

^d^GDS: Geriatric Depression Scale.

^e^BSI: Brief Symptom Inventory.

^f^GDRS: Geriatric Depression Rating Scale.

^g^NR: not reported.

^h^BDI-SF: Beck Depression Inventory short form.

^i^SDS: Zung Self-Rating Depression Scale.

### Summary of the Evidence

Table 3 is the summary of evidence table recommended by JBI [12]. It shows the 4 most commonly reported outcomes for each review paper. Parra-Vidales et al [37] reported 7 studies but discussed the intervention content of 5. Carer depression improved in 71% (5/7) of the meta-analyses, and anxiety improved significantly in 75% (3/4) of the meta-analyses. The outcomes for caregiver burden were inconsistent; 20% (1/5) reported improvement, 60% (3/5) found no effect or improvements in the control groups, and 20% (1/5) had mixed results. Self-efficacy was investigated in 1 meta-analysis [15], which found positive results. A total of 5 reviews stated that the personalization of interventions was important [15,16,28,38,42]. In total, 3 reviews suggested a need for further research into the intervention effects for specific population groups, such as caring for people with different types of dementia or the nature of the relationships with the person with dementia (spouse, child, and sibling) [24,25,39]. Multicomponent interventions that combined telephone and internet delivery methods with elements of education, psychotherapy, and social support had the largest reported effect sizes [24,25,29,38]. However, most review papers did not differentiate the results for each type of intervention or delivery method.

**Table 3:** Summary of intervention content, delivery, and results of the 4 most commonly measured outcomes.

Study	Intervention content, n (%)					Intervention delivery, n (%)					Depression^a^		Anxiety^a^		Burden^a^		Self-efficacy^a^
	Educational	Psychotherapeutic	Social	Multicomponent	Telephone		DVD	Computer	Mixed								
Leng et al, 2020 [15]^b^	9 (52.9)	1 (5.9)	0 (0)	7 (41.2)	0 (0)		0 (0)	14 (82.4)	3 (17.6)	+		+		–		+	
Deeken et al, 2019 [24]^b^	6 (18.2)	7 (21.2)	2 (6.1)	18 (54.5)	11 (33.3)		1 (3)	11 (33.3)	10 (30.3)	+		NR^c^		+		NR	
Hopwood et al, 2018 [28]	8 (20)	0 (0)	2 (5)	30 (75)	0 (0)		0 (0)	39 (97.5)	1 (2.5)	+		+		+		+	
Boots et al, 2014 [38]	3 (25)	0 (0)	0 (0)	9 (75)	0 (0)		0 (0)	9 (75)	3 (25)	+		–		?		+	
Egan et al, 2018 [31]	4 (50)	1 (12.5)	0 (0)	3 (37.5)	0 (0)		0 (0)	6 (75)	2 (25)	?		+		–		+	
Jackson et al, 2016 [25]	6 (27.3)	3 (13.6)	2 (9.1)	11 (50.0)	13 (59.1)		0 (0)	5 (22.7)	4 (18.2)	?		+		+		+	
Lucero et al, 2019 [26]	0 (0)	0 (0)	0 (0)	12 (100)	6 (50)		1 (8.3)	2 (16.7)	3 (25)	?		?		?		?	
Pleasant et al, 2020 [16]	18 (94.7)	0 (0)	0 (0)	1 (5.3)	0 (0)		0 (0)	19 (100)	0 (0)	+		+		?		?	
Scott et al, 2016 [36]^b^	0 (0)	0 (0)	0 (0)	4 (100)	0 (0)		2 (50)	2 (50)	0 (0)	+		NR		NR		NR	
Waller et al, 2017 [27]	0 (0)	12 (35.3)	3 (8.8)	19 (55.9)	15 (44.1)		0 (0)	10 (29.4)	9 (26.5)	?		?		?		?	
Zhao et al, 2019 [30]^b^	2 (33.3)	0 (0)	0 (0)	4 (66.7)	0 (0)		0 (0)	4 (66.7)	2 (33.3)	+		+		–		NR	
Etxeberria et al, 2020 [29]^b^	4 (44.4)	0 (0)	0 (0)	5 (55.6)	0 (0)		0 (0)	9 (100)	0 (0)	+		–		–		NR	
Frias et al, 2020 [22]	4 (50)	4 (50)	0 (0)	0 (0)	3 (37.5)		0 (0)	3 (37.5)	2 (22.2)	?		?		?		+	
Godwin et al, 2013 [32]	1 (12.5)	0 (0)	0 (0)	7 (87.5)	4 (50)		0 (0)	3 (37.5)	1 (12.5)	+		+		+		+	
Kishita et al, 2018 [23]^b^	4 (44.4)	5 (55.6)	0 (0)	0 (0)	4 (44.4)		2 (22.2)	2 (22.2)	1 (11.1)	?		+		?		NR	
Klimova et al, 2019 [33]	4 (66.7)	1 (16.7)	0 (0)	1 (16.7)	0 (0)		0 (0)	6 (100)	0 (0)	+		NR		NR		NR	
Parra-Vidales et al, 2017 [37]	5 (71.4)	0 (0)	0 (0)	2 (28.6)	0 (0)		0 (0)	6 (85.7)	1 (14.3)	+		+		NR		?	
Powell et al, 2008 [39]	0 (0)	2 (13.3)	0 (0)	13 (86.7)	2 (13.3)		0 (0)	11 (73.3)	2 (13.3)	?		?		?		+	
Lee, 2015 [34]	0 (0)	0 (0)	5 (100)	0 (0)	0 (0)		1 (20.0)	3 (60.0)	1 (20.0)	+		NR		?		+	
Thompson et al, 2007 [21]^b^	0 (0)	0 (0)	0 (0)	4 (100)	2 (50)		0 (0)	1 (25)	1 (25)	–		NR		NR		NR	
McKechnie et al, 2014 [35]	0 (0)	8 (57.1)	0 (0)	6 (42.9)	0 (0)		1 (7.1)	11 (78.6)	2 (14.3)	?		+		+		?	

^a^+ indicates an improvement, ? indicates mixed results, and – indicates no significant improvement or improvements in the control group.

^b^The papers are the results from meta-analyses rather than the results from individual studies.

^c^NR: not reported.

### Conclusions of Review Papers

The overall conclusion from the review papers was that technology-based interventions were effective in helping informal carers of persons with dementia [22], but there were difficulties in comparing the effectiveness because of variations in study methods (Multimedia Appendix 3 [15,16,21-39]). Some studies have suggested that rigorous and standardized methods are needed to enable effective comparisons between interventions [30-32]. Low-quality RCTs have been reported, and suggestions for future research included the use of high-quality studies [22,27,29,38].

Another suggestion from the review papers was that the lack of adherence to interventions by carers needs further investigation, with many studies not reporting on adherence, attrition bias, and poor response rates [27,37]. To overcome this, feedback from carers regarding the acceptability of the intervention could be collected. Regarding carer characteristics, 10% (2/21) of the review papers suggested further exploration of the impact of technology-based interventions for carers who look after persons with dementia at different stages of dementia or with differing dementia types [25,28]. It has also been suggested that the long-term effects of interventions should be assessed by collecting data at more time points [16,36] and after booster sessions [16]. Training on how to use the technology may also be useful so that those unfamiliar with it are not discouraged from using the intervention [33].

### Findings of This Review

The first objective of this review was to identify the types of interventions that have been developed for informal carers of persons with dementia and to report on their effectiveness. The intervention content was classified into 4 categories: psychoeducational, psychotherapeutic, social, and multicomponent. The delivery of “technology-based interventions” was classified into the categories of telephone, web-based, DVD, and mixed.

The second aim was to report the most effective type of web-based intervention. The findings suggest that multicomponent interventions were the most effective, especially when they used mixed delivery methods, such as telephone and computer. The 3 review papers that included technology-based interventions as well as face-to-face interventions reported that technology-based interventions were just as effective as face-to-face interventions [21-23].

## Discussion

### Principal Findings

Web-based interventions and services (eHealth) are a rapidly growing area of health care delivery. This study identified and synthesized findings from 21 systematic review papers that examined the effectiveness of web-based interventions to support informal carers of persons with dementia. Web-based interventions were evaluated in 119 studies across the reviews. The delivery methods and intervention content varied between interventions, but the most effective interventions were mixed delivery and mixed content.

### Clinical Findings

The findings of this review suggest that technology-based interventions are beneficial in supporting informal carers of persons with dementia. However, several points need to be considered by clinicians before recommending and implementing an intervention.

Several reviews suggested that personalization of interventions to specific carer groups was beneficial, including differentiating between carers caring for patients at different stages of dementia, carers caring for patients with different types of dementia, and carers with different levels of experience [15,16,28,31,38]. Leng et al [15] reported that a questionnaire asking informal carers about their time available to participate and the severity of the dementia of the person they are caring for allowed interventions to be customized for the individual situation.

The delivery method is important to consider. Mixed delivery methods showed greater improvements than web-based, telephone, or DVD-based interventions alone. This could be because of the reported lack of adherence to web-based interventions that do not involve personal contact with the clinicians [27,37]. The personalized contact in mixed delivery interventions could support participants to continue with the intervention using “live” telephone calls by clarifying queries arising from web-based materials and addressing other concerns that arise [7].

Although multiple review papers have analyzed research in this field to date, there has been no umbrella review. One of the unique aspects of an umbrella review is that it considers the overlap of studies included in other review papers. It is thus able to review when data are being reported multiple times and synthesize the data, giving a clearer insight into the effectiveness of, in this paper, the eHealth interventions. Of the 119 studies included in the review, 50 (42%) were reported more than once. The results from the studies by Kajiyama et al [44] and Beauchamp et al [45] were included in 12 of the reviews, which could give an unbalanced and unwarranted emphasis on their findings. An umbrella review considers these factors and offers clinicians an integrated review where these factors are considered. This is the first umbrella review on this topic, and therefore, it has important clinical and research implications.

### Strengths and Limitations

The differences in intervention content affected comparisons of data, with many reviews reporting their findings descriptively rather than pooling the diverse statistical data. The quality scores may also have been skewed by the heterogeneous methods in many reviews, resulting in a lack of meta-analyses. A total of 2 of the critical AMSTAR 2 items [17] assessed whether a meta-analysis was conducted; therefore, a negative response to those items affected the results. Further supporting this point, the only 2 reviews to score as high quality contained meta-analyses [15,24]. This may be a limitation of AMSTAR 2, leading to downgrading reviews that were otherwise well conducted but could not conduct a meta-analysis owing to the heterogeneity of the data. AMSTAR 2 was selected over the JBI recommended critical appraisal checklist [12] as it is widely recommended to assess the quality of systematic reviews [46].

The CCA score was used to ascertain the overlap of the studies included in the reviews. A measure of overlap was selected owing to the presence of 1 study [44] in 12 of the reviews [15,16,22,24-26,28,30,31,33,36,37]. It is suggested that CCA can be misrepresentative of the true overlap by the inclusion of several “index publications.” The overall moderate score of 7% overlap, yet the high scores of comparisons between individual papers, suggests that the overall score may have been skewed by a high number of “index publications” [20]. The high overlap between several review papers could mean that despite “new” systematic reviews being conducted, there is a false impression of new evidence. This could mask the lack of research on this topic.

A limitation of reviewing the reviews is that the data were limited to what the previous authors reported on. The results were not separated for different intervention types, formal or informal carers, and delivery methods, making it difficult to conclude on the most effective intervention type. Standardized and more scientifically rigorous methods have been suggested to enable better comparison, so development of more effective interventions can occur [30-32].

Demographic data, such as age or dementia type, were not reported in many of the reviews. This could be because the studies themselves did not report the data, thus limiting the review papers. The countries in which the studies were conducted were all high-income countries, showing a potential lack of research in low-income countries. Rural countries may benefit the most from web-based interventions as the support can be provided to carers from anywhere. However, these interventions assume that carers are able to read, have an internet connection, and access a computer.

### Conclusions and Recommendations

The findings of this review demonstrated the use of a wide variety of intervention methods and delivery, content, and outcome measures when studying web-based interventions for carers of people with dementia, making comparisons across studies difficult. Web-based interventions showed most positive results for improving carers’ depression and anxiety; however, other outcomes were not consistent. Interventions that included psychoeducation, psychosocial, and psychotherapeutic elements were the most effective in improving carer well-being. Tailored interventions for the individual to ensure relevance also improved effectiveness. Nevertheless, the methodological quality of many of the review papers was “critically low,” and this needs to be considered when interpreting the results.

Web-based interventions have the potential to reach informal carers of persons with dementia in geographically isolated areas where support may be difficult to access. Further research should aim to distinguish between the types of technology-based intervention content and delivery, thus enabling easier comparison of results. Other suggestions include a focus on specific groups of carers, such as those with different relationships with persons with dementia and those caring for patients with varying stages and types of dementia, which could help personalize the interventions and potentially encourage carers to continue to use the interventions. Research focusing on specific populations or intervention types will facilitate the development of effective web-based interventions in the future.

This is the first umbrella review to examine the effectiveness of technology-based interventions for informal carers of persons with dementia. Previous research has noted the effectiveness of multicomponent interventions and the importance of personalizing interventions. However, the overlap of research on this topic has not been reported previously. The overlap of research on the effectiveness of eHealth interventions for informal carers of persons with dementia may lead to this topic being neglected, and this has consequences for future research.

## Appendix

Multimedia Appendix 1 Corrected covered area citation matrix and individual overlap percentages between each of the review papers.

Multimedia Appendix 2 Characteristics of the review papers.

Multimedia Appendix 3 Aims, results, and conclusions of the review papers.

## Abbreviations

AMSTAR: A Measurement Tool to Assess Systematic Reviews
CCA: corrected covered area
ESRC: Economic and Social Research Council
JBI: Joanna Briggs Institute
RCT: randomized controlled trial
