# Supplementary material for: eHealth and Web-Based Interventions for Informal Carers of People With Dementia in the Community: Umbrella Review
Source: J Med Internet Res. 2022 Jul 22;24(7):e36727. doi: 10.2196/36727 (PMC9356334; doi:10.2196/36727)
Supplement: Multimedia Appendix 2 [file jmir_v24i7e36727_app2.docx]

Appendix B. Characteristics of included studies.

| Authors | Countries of studies | Number of papers reviewed | Population | Sample sizes | Type of study included | AMSTAR score |
| --- | --- | --- | --- | --- | --- | --- |
| Boots et al. (2013) | Not reported | 12 studies (1995-2013) | 11/12 studies reported mainly female carers and 1/12 did not report on gender. 5/12 included mainly children caring for a parent, 4/12 were spouses and 3/12 did not report on the relationship. | 7 papers had no/ did not report a control group (N= 1033, range 21-700+) Intervention N=265, range 3-150; control N=251, range 8-149 | 3 RCTs, 4 mixed methods, 1 single-group pre-test/post-test, 1 quasi experimental, 2 multiple-group pre-test/ post-test, 1 formative evaluation | Moderate |
| Deeken et al. (2019) | Countries not reported, participants were described as American and Cuban American carers (n=1), Chinese American carers (n=1), living in rural Pan-handle area of Florida (n=1), African America carers (n=1), carers veterans (n=2), | 33 studies (1990-2017) | Mean age is 62 yrs. (range 18-85) but not all studies reported age. Mainly female participants and 4/33 were female only and 1/33 was male only. | Total n= 3313, range 11-299; intervention/ control group stats were not reported | RCTs | High |
| Egan et al. (2017) | USA (n=3), Netherlands (n=1), France (n=1), UK and Netherlands (n=1), Canada (n=1), China (n=1) | 8 studies (1995-2015) | No demographic information is presented (e.g. age, gender, ethnicity). Populations are described as employed family carers of PWD; carers of PWD; carers of people with AD; Dementia caregivers (both laypeople and professionals with subgroup results presented); family carers; older adults with neurodegenerative disease with AD subgroup. | Intervention N=504 (range 3-151); control N=462 (range 8-149). | RCTs | Moderate |
| Etxeberria et al. (2020) | Not reported | 10 studies (2014-2018) | The mean age ranged from 53-70. 8/10 reported on gender, most participants were female. 5/10 gave percentage ranges for the number of spouses and 2/10 reported on the number of children. 1/10 described the participants as Hispanic. 2/10 included professionals as well as family carers. | Intervention N=619, range 17-149; control N=499, range 23-122 | 6 RCTs, 2 quasi-experimental with no randomisation | Low |
| Frias et al. (2019) | China (n=2), Netherlands (n=1), USA (n=5) | 18 studies (2005-2016)- 8 technology based | No demographic information presented from the included studies (e.g. age, gender, ethnicity). The authors note the focus is on “the main family caregiver”. | Intervention N=1559, range 16-323; control N=1533, range 17-319 | RCTs | Low |
| Godwin et al. (2013) | Not reported | 8 articles of 4 studies (1995-2007) | Carers of people with “AD or related dementias”. The average age (7 papers gave demographics information) was 60.9 and participants were 71% female and 74% white (5 papers). | 46-1222 | RCTs | Critically low |
| Hopwood et al. (2018) | UK (n=1), USA (n=18), Netherlands (n=9), Canada (n=5), France (n=1), Poland (n=1), Germany (n=2), China (n=2), multinational; Spain, Poland, Denmark (n=1) | 40 studies (1995-2018) | Mean age from 30/40 was 60.2. 1/40 reported gender and included female carers. 3/40 included professionals and informal carers. Two of those involved PwD as well. 4/40 looked at carers of veterans, 2/40 specified Hispanic carers, 1/40 was Chinese carers and 1/40 included Spanish speaking carers. The type of dementia was referred to as dementia in 30/40. 4/40 specified AD, 3/40 neurodegenerative disease, 1/40 MCI, 1/40 “impaired cognition” and 1/40 did not report the type of dementia. | Total participants N= 3060, range 4-315. Control group stats were not reported. | 9 RCTs, 7 quasi-experimental studies, 4 qualitative studies, 20 with mixed or other methods | Moderate |
| Jackson et al. (2016) | Netherlands (n=1), USA (n=16), Canada (n=1), China (n=2), multinational- UK, Spain, Greece (n=1), Germany (n=1) | 22 studies (1998-2015) | All dementias, AD, MCI, VD or mixed dementia, unpaid informal carers. Two studies included mainly African American, Cuban American, and Hispanic American carers. One study included carers of army veterans. | 11 papers had no/ did not report a control group (N= 1815) Intervention N=727, range 7-149; control N=594, range 7-117 | 16 RCTs, 3 within subjects baseline treatment, 2 pre/post-test designs, 1 mixed methods | Moderate |
| Kishita et al. (2018) | France (n=1), Australia (n=1), US (n=5), Netherlands (n=1), China (n=1) | 30 studies (2006-2016)- 9 technology based | Carers of people with AD (n=1), dementia (n=4), mixed- including AD, VD or FtD (n=4) | Not specified | RCTs | Critically low |
| Klimova et al. (2019) | China (n=1), USA (n=1), multinational- Australia, Denmark, Netherlands, UK, Spain, and Poland (n=4) | 6 studies (2013-2018) | 3/6 reported on participants age (range: 25-88). 5/6 involved only ICs and 1/6 had both formal and informal. 2/6 included information on participants gender of these, 73.4% of participants were female. The type of dementia was not mentioned. | Total n= 755, range 35-279; intervention/ control group were reported by one study. Intervention group n=30, control n=31 | 3 RCTs, 2 survey quality studies, 1 experimental study | Critically low |
| Lee (2015) | The results section states that most participants were carers in the US or Canada | 5 studies (2003-2011) | 4/5 reported a mean age = 66.7. 3/5 described participants as carers of PwD. 1/5 included carers of people with AD. 1/5 included carers of people with neurodegenerative disorders such as AD, VD or Parkinson’s disease. Gender and relationship to the person with dementia was not reported. | Intervention/ control group stats not reported. Total N= 412, range= 28-127 | Quasi-experimental and experimental | Critically low |
| Leng et al. (2020) | USA (N=8), Netherlands (n=3), France (n=2), Canada (n=1), Germany (n=1), Spain (n=1), UK (n-1) | 17 studies, 14 in the meta-analysis (1995-2019) | Demographics not reported- inclusion criteria states that participants were unpaid family carers such as a spouse or adult child currently caring for a family member with dementia. | Intervention N=1208, range 14-357; control N= 994, range 11-190 | RCTs | High |
| Lucero et al. (2019) | Not reported | 12 studies (1995-2015) | Not all studies reported on participant gender or ethnicity. Most participants were female and where race was reported, participants were mainly Caucasian. One study looked at male caregivers and another included Chinese American caregivers. | Total N= 1453, range 32-250. Intervention/ control groups not reported. | RCTs | Moderate |
| McKechnie et al. (2014) | US (n=7), Canada (n=3), Sweden (n=1), Norway (n=1), Netherlands (n=1), not specified (n=1) | 14 studies (2003-2011) | 8/14 described participants as family carers and 1/14 specified spousal carers.5/14 involved carers of people with an unspecified dementia, 1/14 was AD, 2/14 specified AD and related disorders, 1/14 looked at neurodegenerative diseases, 2/14 included carers of people with dementia or having had a stroke and 3/14 did not report on the type of dementia. Participant gender and age were not reported. | Intervention/ control group stats not reported. Total N= 1165, range= 18-329 | 6 RCTs, 1 controlled trial, 4 single-group pre-test/post-test, 2 single-group post-test, 1 multiple-group pre-test/post-test | Critically low |
| Parra-Vidales et al. (2017) | Not reported | 7 studies (2010-2015) | 6/7 studies included informal carers. 1/7 did not specify whether they were informal or not. 1/7 specified a diagnosis of AD. 4/7 included unspecified dementias.1/7 included carers of people with a functional/ cognitive impairment. 1/7 reported a mixture of people caring for those with AD, VD, or other dementias. Age, gender, and relationship to the person with dementia was not reported. | 4/7 reported intervention/ control group statistics. Intervention N=117, range 3-75; control N=121, range 8-75. 3/7 did not report/ had no control group, total N=164 | 4 experimental with control group, 1 qualitative no control group, 1 experimental with no control group, 1 iterative process in the development of content | Critically low |
| Pleasant et al. (2020) | USA (n=12), Netherlands (n=2), France (n=1), Canada (n=1), multinational- Netherlands and UK (n=1), Not reported (n=1) | 18 articles, 19 studies (2002-2016) | Included studies look at informal carers (n=9), formal (n=7) and both (n=2). Mainly female (14/19) and Caucasian (8/19) sample, but 4/19 reported a sample of ethnic minority, 4/19 mainly children, 4/19 mainly spouses. Average ages range from 21-78. Educated to high school level (4/19), college (6/19), university (2/19) or not specified (6/19) | 10 papers had no/ did not report a control group (N= 503) Intervention N=590, range 14-150; control N=675, range 14-209 | 8 RCTs, 9 pre/post cohort design, 2 pre/post cohort design with control groups | Moderate |
| Powell et al. (2008) | Authors state that all studies were conducted in North America | 15 articles, 5 interventions (1995-2007) | Informal carers of PwD in the community | Intervention N=234, range 23-77; control N=305, range 23-75. One intervention (2 studies) only had an intervention group (n=21). Multiple papers on the same interventions were reported as using the same participants. | 13 RCTs, 2 pre-test/post-test studies | Critically low |
| Scott et al. (2016) | Not reported | 4 studies (2000-2013) | 80% of participants were female. 35.5% were caring for their spouse and 53.2% were caring for a parent. Studies looked at those caring for a person with dementia (n=2) or a combination of people with AD, vascular dementia or “other” (n=2). | Total n= 505, range 33-299; intervention/ control group stats were not reported. | 2 RCTs, 2 waitlist control trials | Moderate |
| Thompson et al. (2007) | Not reported | 44 studies, 4 technology based (1990-2003) | Not reported | Intervention N=287, range 51-152; control N=206, range 31-73 | RCTs | Critically low |
| Waller et al. (2017) | USA (n=20), China (n=3), Canada (n=2), UK (n=1), Netherlands (n=4), Germany (n=2), France (n=1), Spain (n=1) | 34 studies (1990-2016) | Primary caregivers living with a PwD. Studies looking at specific groups included spouse carers (n=1), care recipient in a nursing home (n=1), female carers only (n=2), spouse carers of veterans (n=1), | Total n= 3851, range 11-299; intervention/ control group stats were not reported | 30 RCTs, 1 cluster randomised controlled trial, 4 NRCTs | Moderate |
| Zhao et al. (2019) | Not reported | 6 studies (2005-2015) | Mean age is 58.66 yrs., 4/6 studies reported gender: 73.5% were female. Participants were described as caregivers of PwD or potential memory loss | 3/6 reported intervention/ control stats. Intervention n= 232, range 25-150; control n=219, range 24-149. 3/6 reported no intervention/ control stats. Total n= 401, range 60-245 | RCTs | Moderate |
