# Supplementary material for: eHealth and Web-Based Interventions for Informal Carers of People With Dementia in the Community: Umbrella Review
Source: J Med Internet Res. 2022 Jul 22;24(7):e36727. doi: 10.2196/36727 (PMC9356334; doi:10.2196/36727)
Supplement: Multimedia Appendix 3 [file jmir_v24i7e36727_app3.docx]

Multimedia Appendix 3. Aims, Outcomes, and Conclusions of Included Review Articles

| Authors | Aims | Outcomes | Conclusions |  |  |  |
| --- | --- | --- | --- | --- | --- | --- |
|  |  |  |  |  |  |  |
| Leng et al. 2020 | Assess the effectiveness of online supportive interventions on informal carer wellbeing and potential effects on the care recipient. | Reductions in depressive symptoms, perceived stress, anxiety and self-efficacy but not burden, coping, QoL or caregiver reactions to behavioural symptoms. Personalization through questionnaire and tailored info from experts was most effective. | Six studies showed benefits on QoL and neuropsychiatric symptoms in care recipients. |  |  |  |
| Deeken et al. 2019 | Estimate the efficacy of tech-based interventions for informal carers of people with dementia. | Small but significant effect of tech-based interventions on carer depression and burden. Multicomponent interventions showed the best results. | Interventions are flexible and available so offer a promising alternative to traditional services. More RCT are needed for specific carer groups. |  |  |  |
| Hopwood et al. 2018 | Identify key components of online interventions for family dementia carers, which components are valued by carers and consider the effectiveness of these interventions. | Most studies show positive results for depression, anxiety, and burden with particular benefit from online social support. Education was best when tailored for the individual, online professional advice was valued by carers. | Mainly positive results, high-quality studies are needed to determine effectiveness with a focus on supporting carers at different stages of dementia. |  |  |  |
| Boots et al. 2013 | Overview of evidence for effectiveness, feasibility, and quality of online interventions of informal dementia caregivers. | Online interventions can improve confidence, depression and self-efficacy when tailored to the individual and are multicomponent. Social interactions with coaches or peers may also be beneficial. | Online interventions for informal dementia carers lack evidence of a high methodological quality. |  |  |  |
| Egan et al. 2018 | Review rapidly growing evidence base for online training and support programs which show potential for scaling up. | Inconsistent outcomes due to methodological diversity. | Standardise methods to enable better comparison and development. None looked at quality of care. |  |  |  |
| Jackson et al. 2016 | Assess effectiveness of interventions delivered by phone, online or both for carers of people with AD, VD, or mixed dementia in the community. | Multicomponent interventions were better than just internet or phone for reducing depression, burden, and self-efficacy. | Further research to target specific dementia types needed. |  |  |  |
| Lucero et al. 2019 | Evaluate the state of the science of ICT interventions on health of informal dementia carers. | Telephone interventions have the largest reductions in depression, anxiety, and burden. | Limited statistically significant results for ICT interventions, further research needed to generalise and validate ICT interventions. |  |  |  |
| Pleasant et al. 2020 | Describe the literature on dementia based online learning, identify gaps, and present recommendations to create training programs that are satisfactory to both formal and informal caregivers. | Online training programs are more efficient and convenient than live training. Trainers should personalise these programs. Improvements were seen in knowledge, self-efficacy, anxiety, depression, burden, and satisfaction but not consistently in care competency, stress, and care recipient status. | More online training programs are needed, measured at more time points with booster sessions and control groups. |  |  |  |
| Scott et al. 2015 | To report on the effectiveness of pure tech-based CBT interventions for dementia carers. | Tech-based CBT has similar depression reduction to face to face CBT however only 4 studies were analysed. | Further research into long term efficacy and the underpinning mechanisms of these programs is needed to provide more targeted interventions. |  |  |  |
| Waller et al. 2017 | To assess the scope, volume, and quality of research on the acceptability, utilisation and effectiveness of telephone and computer-based interventions for caregivers of people with dementia. | Psychoeducation, peer-support, skills training, and health assessments were beneficial to carer wellbeing. | Interventions that give practical care strategies, wellbeing advice and social support need high quality trials to determine effectiveness. Use of computer-based interventions decreases over time. |  |  |  |
| Zhao et al. 2019 | To examine the effect of online interventions on mental health outcomes of family caregivers of PwD and explore which components play an important role. | Online interventions are generally effective at reducing anxiety and depression in dementia carers, burden and stress need more investigation. | A standardised assessment tool is needed to compare the interventions effectively and keep up with the constant new developments in tech-based interventions. |  |  |  |
| Etxeberria et al. 2020 | Analyse the effectiveness of online support programs and whether they enhance informal dementia carers wellbeing. | Improvements in depression, anxiety, burden, and caregiving competence but only depression was significant. Largest effects were made up of multicomponent interventions. | Rigorous methodological criteria are needed in further research to demonstrate effectiveness. |  |  |  |
| Frias et al. 2019 | Assess effectiveness of psychoeducational interventions on family caregivers of PwD living at home. | Positive results for tech-based interventions and burden, in all but one study. | More rigorous methodology needed but tech-based follow up is cost effective and effective. |  |  |  |
| Godwin et al. 2013 | Assess the psychosocial effects of tech-driven interventions for informal dementia carers. | Variance of measures led to inconsistent results. Depression and anxiety decreased, strain had conflicting outcomes, social support was measured with different instruments in every study. | Positive findings but, variance in measures and content meant comparison is difficult. Standardised methods are needed. |  |  |  |
| Kishita et al. 2018 | Updating literature on interventions for dementia carers and efficacy on mental health outcomes. | Psychoeducation and skill building reduce burden. CBT based psychotherapeutic interventions reduce anxiety and depression. Delivery mode does not seem to impact effectiveness of interventions but attrition rates for technology based is lower than face-to-face interventions. | More research is needed to test efficacy of modern forms of acceptance and commitment therapy. |  |  |  |
| Klimova et al. 2019 | Consider the benefits and limitations of e-learning as a support for informal dementia carers. The implications to the care and the carers. | Educational programs help carers feel more confident about dementia care, reduce stress, and enhance feelings of empathy. | Carers need training in how to use the technology so older carers aren't put off by the tech side. |  |  |  |
| Parra-Vidales et al. 2017 | Review online interventions for dementia carers which are meant to be effective in improving QoL and reducing incidences of disease associated with caring. | Psychoeducation improves self-efficacy, anxiety, and depression. | Lack of adherence interventions over time needs further investigation. |  |  |  |
|  |  |  |  |  |  |  |
| Powell et al. 2008 | Report on the effectiveness of networked ICT interventions in supporting dementia carers. | Inconsistent outcomes but moderate effects for multicomponent interventions on stress and depression. Effects varied with caregiver ethnicity, formal support, and baseline depression. | Further evaluation of the role of ICT in supporting informal carers is needed in robust trials with follow ups. |  |  |  |
|  |  |  |  |  |  |  |
| Lee 2015 | Investigate the effectiveness of technology-based social support groups for burden in carers of PwD. | Overall burden scores showed no significant change but wives caring for their partners, frequent users compared to occasional users and those with lower levels of mastery showed significant improvements. Stress reductions were significant and there were reductions in depression and “troubling dementia-related behaviours”. | Bilingual services are important to make interventions accessible for all carers. The feasibility for older caregivers to use the technology is a concern. Online support groups demonstrate small but significant outcomes, seem to be cost- effective while reducing burden in carers of PwD. |  |  |  |
| Thompson et al. 2007 | Investigate the effects of information and support interventions on the quality of life of those caring for a PwD. | The main effects were not significant, but subgroup analyses were. Participants with low-medium mastery scores at baseline showed significant improvements for anxiety, depression, and reaction to behaviours. Wives caring for their husband showed significant reductions in depression. Only four studies used technology-based interventions and they found no significant changes to depression. | The authors conclude that the effectiveness of educational interventions varies greatly and there is a need for more rigorous methods. Suggestions for further research include looking at different groups of carers and when in the dementia journey is best to introduce the programmes to carers. |  |  |  |
| McKechnie et al. 2014 | Evaluating the effects of computer-mediated interventions for informal carers of PwD. | Most of the higher quality studies found significant effects on depression, stress, and burden. Changes to anxiety were found in two studies. Self-efficacy and positive aspects of caring were affected in most studies. | Overall findings are positive, studies found decreases in burden, stress, depression and anxiety. Future studies should look at using more rigorous methodology by defining their aims clearly, having adequate statistical power, including control groups and measuring outcomes that are important to carers themselves. |  |  |  |
